# Three-dimensional Interlocking versus conventional miniplates for mandibular fractures fixation near the mental foramen: a split-mouth randomized clinical trial

**DOI:** 10.1186/s12903-026-07799-5

**Published:** 2026-03-29

**Authors:** Ibrahim Mohamed Abdelhamed, Ahmed Ossama Sweedan, Reem M. Ismail, Yehia El-Mahallawy

**Affiliations:** Oral and Maxillofacial Surgery Department, Faculty of Dentistry, Alexandria University, Alexandria, Egypt

**Keywords:** Mental nerve, Mental foramen, Mandibular fracture, Bone plate, 3D interlocking miniplates, Miniplates

## Abstract

**Objectives:**

To evaluate the 3D-Interlocking miniplate (3D-IMP) in the management of mandibular parasymphyseal fractures near the mental foramen (MF), and compare it clinically and radiographically with conventional miniplates (2MPs) in a split-mouth design.

**Materials and methods:**

A total of 12 patients with recent bilateral mandibular fractures in the MF zone were treated with 3D-IMP on one side and with conventional 2MPs on the other side. Clinical evaluations were carried out for 6 postoperative weeks for the assessment of pain, Intra-fragmentary mobility, occlusion, wound healing complications, and an objective mental nerve neurosensory function. Radiographic assessment for the mean bone density was performed immediately after surgery and at 12 postoperative weeks.

**Result:**

By the end of the follow-up period, all cases reported a lack of intra-fragmentary mobility, proper occlusion, a significant reduction in pain intensity scores, and proper wound healing. Objective nerve testing reported a statistically significant improvement across the clinical follow-up period in both sides; however, one patient reported Level C sensation in the 2MPs side (4.17%) at the 6th postoperative week. Both sides showed a significant increase in mean bone density at 12 weeks (*P* < 0.001^*^), with the 3D-IMP side demonstrating a significantly higher bone density (1107.4 ± 142.2 HU) than the 2MPs side (960.9 ± 97.58 HU).

**Conclusion:**

3D Interlocking miniplates proved to be an effective alternative to conventional miniplates for mandibular parasymphyseal fractures near the MF, offering enhanced bone healing and better neurosensory outcomes.

**Trial registration:**

Trial was retrospectively registered at clinicaltrials.gov [*NCT06939010*/2025-4-22].

**Supplementary Information:**

The online version contains supplementary material available at 10.1186/s12903-026-07799-5.

## Introduction

Mandibular fractures account for approximately 36–59% of maxillofacial fractures, making them among the most prevailing facial skeletal injuries due to the prominent and exposed anatomical position of the mandible [1]. Despite the contemporary innovations in the maxillofacial fixation techniques, mandibular fractures near the Mental Foramen (MF) remain particularly challenging to manage compared to other jaw regions. Excessive torsional stresses, changes in bone trajectory, and risk of mental nerve injury are the challenges encountered during the treatment planning for the management of fractures in the mental foramen region [1, 2].

As per the doctrines of Champy for the management of fractures in the parasymphyseal zone using Michelet’s miniplates to achieve a functionally stabilized fixation, the standard internal fixation approach in the management of MF zone fractures involves dual miniplate fixation, in a subapical and along the lower border positions [3]. Although effective, conventional miniplates offer limited three-dimensional (3D) support, which may be inadequate in areas under higher functional loads [4]. In response, 3D-plates were developed, providing multidirectional stability through a quadrangular structure. However, their use near the mental foramen is apparently limited due to the risk of damaging the neurovascular bundle [4, 5]. To address this, 3D-Interlocking miniplates (3D-IMP) were introduced, offering rectangular plate assembly through an intricate basal U-shaped and a coronal linear plate configuration that safely encircles the mental foramen. This quadratic pattern enhances the biomechanical performance while minimizing risk to the mental nerve [6, 7].

Although several studies have compared three-dimensional and conventional miniplate fixation systems for mandibular fractures, objective evaluation of neurosensory recovery of the mental nerve, particularly in fractures involving the mental foramen region, remains insufficiently explored [8]. Furthermore, quantitative radiographic assessment of bone healing has rarely been incorporated in comparative fixation studies. To date, there is a lack of split-mouth randomized clinical trials that simultaneously assess neurosensory outcomes and radiographic bone healing while controlling for patient-related confounding factors [7].

This study aims to compare the radiographic and clinical outcomes of 3D-IMP versus dual miniplate fixation in the MF zone fractures. It is hypothesized that there would be no difference in radiographic performance between 3D-IMP and double miniplate in the management of fractures in the MF zone. The specific aim of the study was to design and implement a split-mouth study. Primary outcomes of the study include neurosensory recovery and bone density analysis of the fracture lines. The secondary outcomes assess pain, stability, wound healing, and occlusion.

## Materials and methods

### Study design

**Fig. 1:**
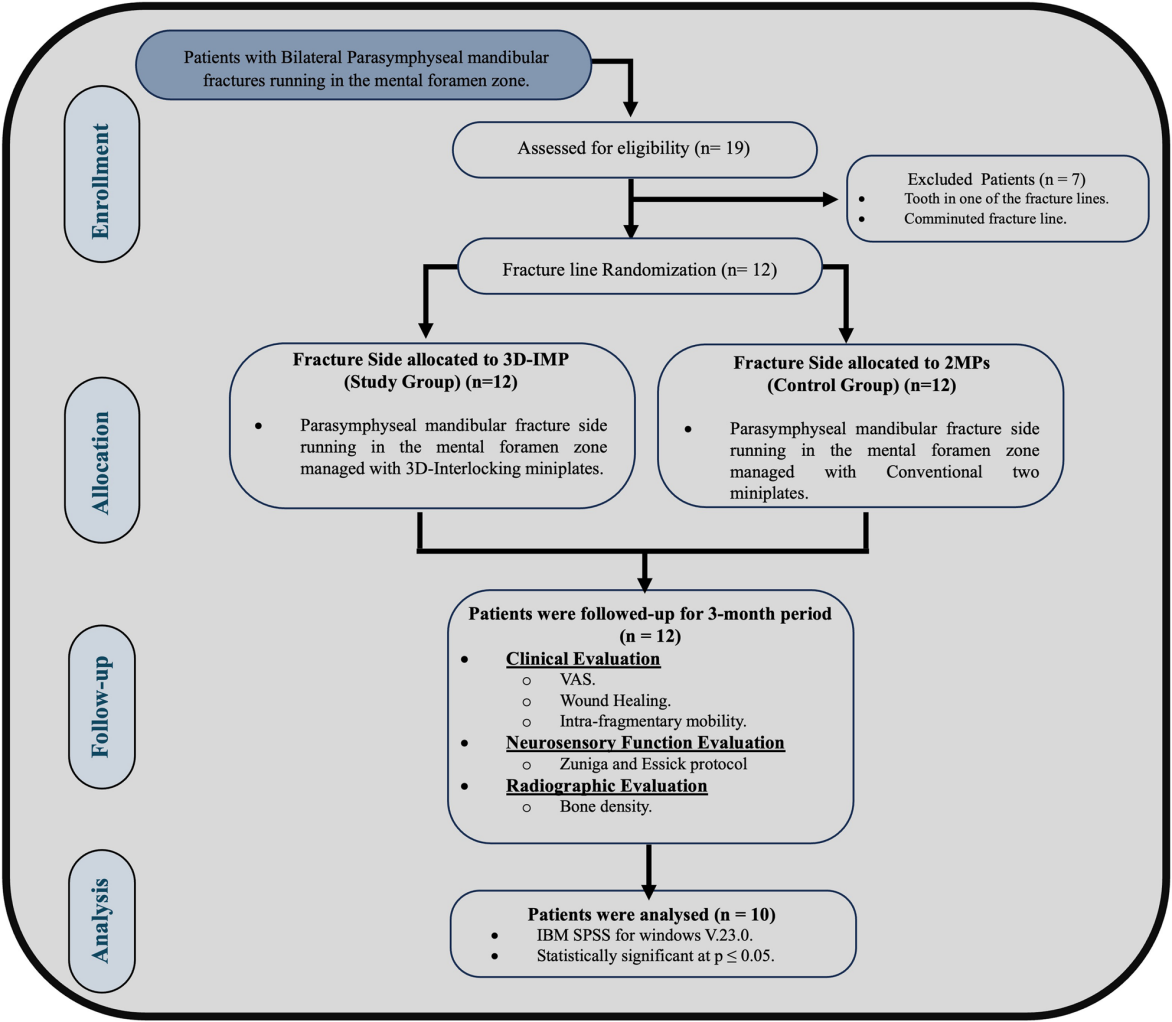
CONSORT Flow chart

This split-mouth randomized controlled trial, adhering to CONSORT guidelines (http://www.consort-statement.org*)*, compared 3D-Interlocking miniplates and conventional double miniplates for the management of fractures in the MF zones (Fig. 1). Sample size estimation was conducted to ensure adequate power for both the primary outcome (radiographic bone density) and secondary clinical outcomes. First, based on the primary outcome of bone density, the sample size was calculated using a confidence interval estimation method. Previous data comparing conventional 2D versus 3D plates indicated a mean difference of 125.14 ± 188.49 [9]. A minimum of 7 pairs was required to achieve the desired precision. A Second sample size estimation was conducted assuming a 95% confidence level and 80% study power for the secondary outcome. The complication rate after 3 months was 70% for the 2D conventional miniplates and 13.33% for the 3D miniplates [4]. Based on comparisons of proportions, a total sample size of 12 patients with bilateral fractures was calculated. To ensure robustness and adequate power for all study parameters, the larger sample size (*P* = 12) was selected. Sample size was based on Rosner’s method, calculated by G*Power 3.1.9.7 [10, 11].

### Patients’ selection

The study included patients with recent bilateral parasymphyseal mandibular fractures running in the mental foramen zone. Adult patients with fractures that required open reduction and internal fixation, without gender predilection, who agreed to follow-up and were medically fit for general anesthesia, were included in this study. In order to test the fixation modality effect on the neurosensory recovery of the open reduction procedure, patients with subjective preoperative neurosensory affection in the course of the mental nerve were excluded from the study. Exclusion criteria also included medically compromised patients, infected fracture lines, pathological or old fractures, completely edentulous patients, and comminuted fractures [6]. Patients provided informed consent outlining the procedures, their benefits, and challenges, yet they were blinded to the fixation system used on each side. They were allocated in a 1:1 ratio using an on-site computer software system with concealed allocation through sequentially numbered, opaque, sealed envelopes (SNOSE). Randomization was conducted with 2 & 4 random block sizes (http://www.randomizer.org/*).* One side was treated with 3D-Interlocking miniplates (3D-IMP), and the other with conventional miniplates (2MPs). Patients were recruited from inpatients admitted to the emergency department at Alexandria University’s teaching hospital from January 2023 to December 2024.

**Fig. 2:**
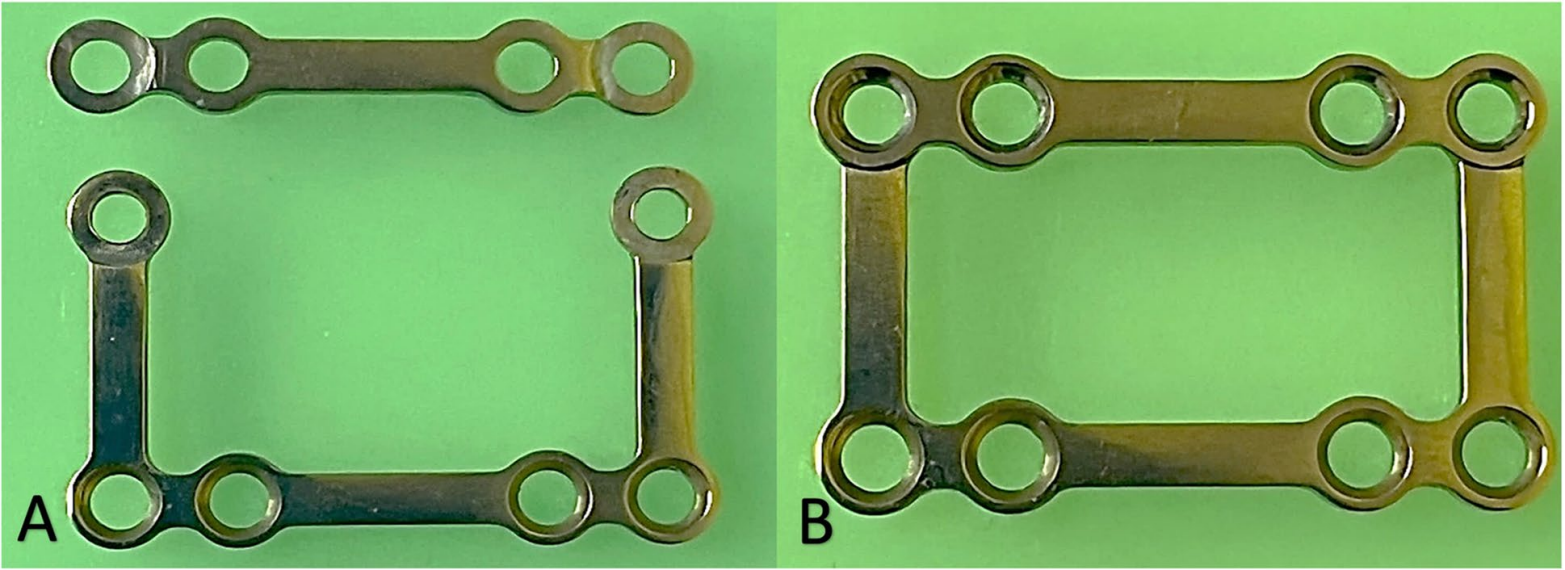
Design of the 3D-Interlocking Plate. **A**, Pre-assembled components: U-shaped base plate and linear plate. **B**, Post-assembly configuration of the 3D Interlocking system. The U-shaped plate exhibits a uniform thickness of 1.5 mm, except at the superior recess, which is milled to 0.75 mm to accommodate the interfacing segment of the linear plate, whose terminal ends are also 0.75 mm in thickness. This precise dimensional adaptation ensures a flush fit upon interlocking, maintaining a consistent total plate thickness of 1.5 mm across the construct

Surgeries were conducted at Alexandria University’s Oral and Maxillofacial Surgery Department under Helsinki guidelines with ethical approval (IRB:00010556-12/2022), and trial registration (retrospectively registered at ClinicalTrials.gov/*NCT06939010*/2025-4-15). The design of 3D-Interlocking miniplates consisted of two plates: a U-shaped plate and a linear plate, both of 1.5 mm thickness. The superior margins of the tissue-contacting surface of the U-shaped plate incorporate circular recesses where the thickness is reduced to 0.75 mm, likewise, the lateral parts of the linear plate have a recess with the same thickness (0.75 mm) in which both plates fit with each other tightly, the thickness of the combined plates at this area becomes 1.5 mm (same as the rest of the plate) (Fig. 2). The vertical strut of the U-shaped plate varies in length from 6 to 9 mm. Upon fixation, the assembled configuration mimics the geometry of conventional 3D miniplates. The titanium 2.0-mm miniplates System is provided with a 1.5-mm Plate profile. Mono-cortical screws were utilized in both groups (Arab Engineers’ co, Cairo, Egypt).

### Preoperative phase

A comprehensive history taking and methodical clinical examination were implemented and logged for all of the enrolled cases. A Computed Tomography (CT) scan was conducted to determine the number and pattern of fracture lines, displacement severity, and the presence of teeth within the fracture line (Philips Brilliance 64 MDCT, Philips, Eindhoven, Netherlands).

### Surgical procedure

Patients underwent the required tests to obtain an anesthesiologist’s clearance. All patients were treated under general anesthesia using nasotracheal intubation. The surgical site was prepared with povidone-iodine scrub solution, followed by draping with sterile towels to expose only the area of surgery. The fracture lines were accessed through a bilateral intraoral vestibular approach, and mobilization of the fractures was performed. Any soft tissue trapped within the fracture lines was removed. Fractures were then reduced to achieve proper anatomical occlusion, with temporary Intermaxillary Fixation (IMF) as a guide for the reduction. The surgical team was constant for all of the enrolled patients in this study, under the supervision of the same main surgeon (I.A). After exposure of the fracture lines, one of the surgical team members was entrusted with side selection and group distribution, by opening the sealed allocation envelope (R.I).

**Fig. 3:**
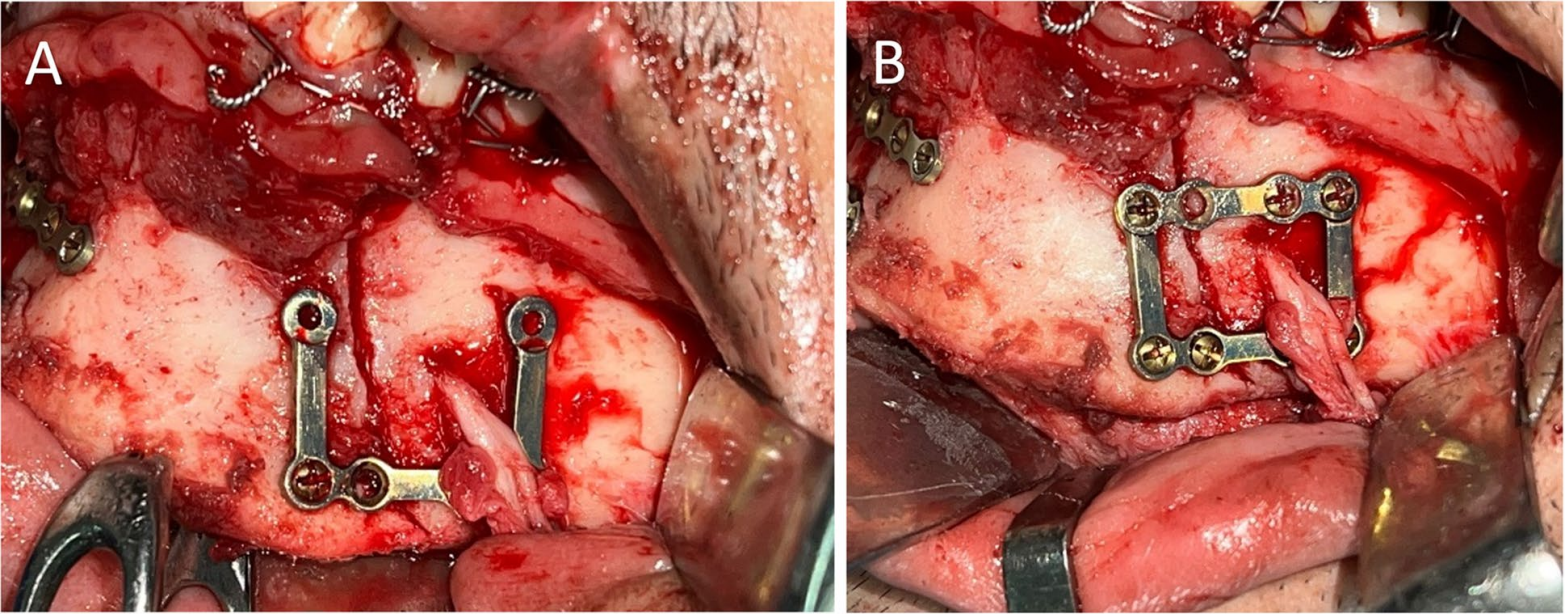
Clinical picture of fracture fixation in the 3D-Interlocking miniplate side. **A**, Fixation of the U-shaped lower border plate. **B**, Fitting the liner upper border plate in the milled recess and fixation using a 2.0 screw

In the *3D-IMP* side, the U-shaped component of the interlocking plate was first positioned across the fracture site, with its vertical bars aligned parallel to the fracture line. The inferior border of the U-shaped plate was fixed using 7-mm screws, which were placed first on both sides of the fracture line to secure the plate (Fig. 3A). Following this, the linear (upper horizontal) component was aligned and secured over the U-shaped plate in the subapical region by engaging the corresponding recesses of both components, ensuring a precise interlock. The superior sub-apical border of the plate was further secured with 7-mm screws. A total of 7–8 screws were utilized in the 3D-IMP side (Fig. 3B).

**Fig. 4:**
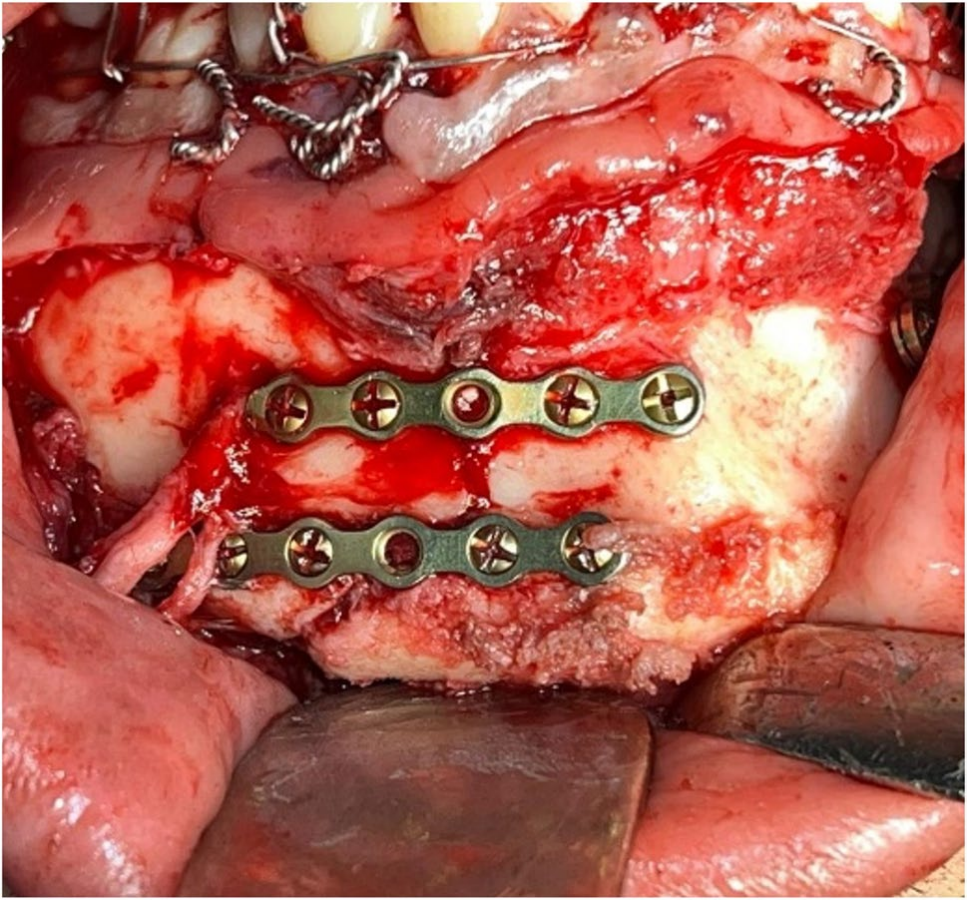
Clinical picture of fracture fixation in the conventional two miniplates side

For the *2MPs* side, two conventional miniplates were applied following Champy’s lines of osteosynthesis and fixed with mono-cortical screws (7-mm screws). The number of screws in the 2MPs side was variable, ranging from 8 to 10 screws (Fig. 4). After bilateral fixation, the transitory IMF was released, and the occlusion was assessed. The surgical wound was meticulously closed in a double-layer manner to promote optimal healing and reduce the potential for postoperative complications. Patients were prescribed 875-mg amoxicillin + 125-mg clavulanic acid b.i.d for 5 days (Augmentin, GlaxoSmithKline, UK). Patients were instructed to apply an ice pack extra-orally for the first 24 h. Additionally, they were advised to adhere to a soft, high-protein, high-calorie diet for four weeks.

### Clinical variables

Clinical outcomes were evaluated through a rigorous follow-up schedule at 24 h, one, four, and six postoperative weeks using several indices. Pain was assessed using the 10-point Visual Analogue Scale (VAS). Intra-fragmentary mobility was evaluated by bimanual palpation at the fracture sites. Postoperative occlusion was qualitatively evaluated (satisfactory or deranged). Wound evaluation was subjectively evaluated by the dichotomous monitoring of the presence/absence of healing complications (Yes/No), such as signs of infection or wound dehiscence. A modified Zuniga and Essick protocol was used to assess neurosensory function through three levels of testing. Level A indicates normal nerve function, which includes Static Light Touch (SLT), Brush Directional Stroke (BDS), and Two-Point Discrimination (TPD), with TPD measured using a vernier caliper set at a 5 mm distance between prongs. Level B assessed proprioception using a 3–0 Prolene suture. Level C indicates response to noxious stimuli through Thermal Discrimination (TD) using a cotton roll soaked in solvent ether is evaluated [8].

### Radiographic variables

**Fig. 5:**
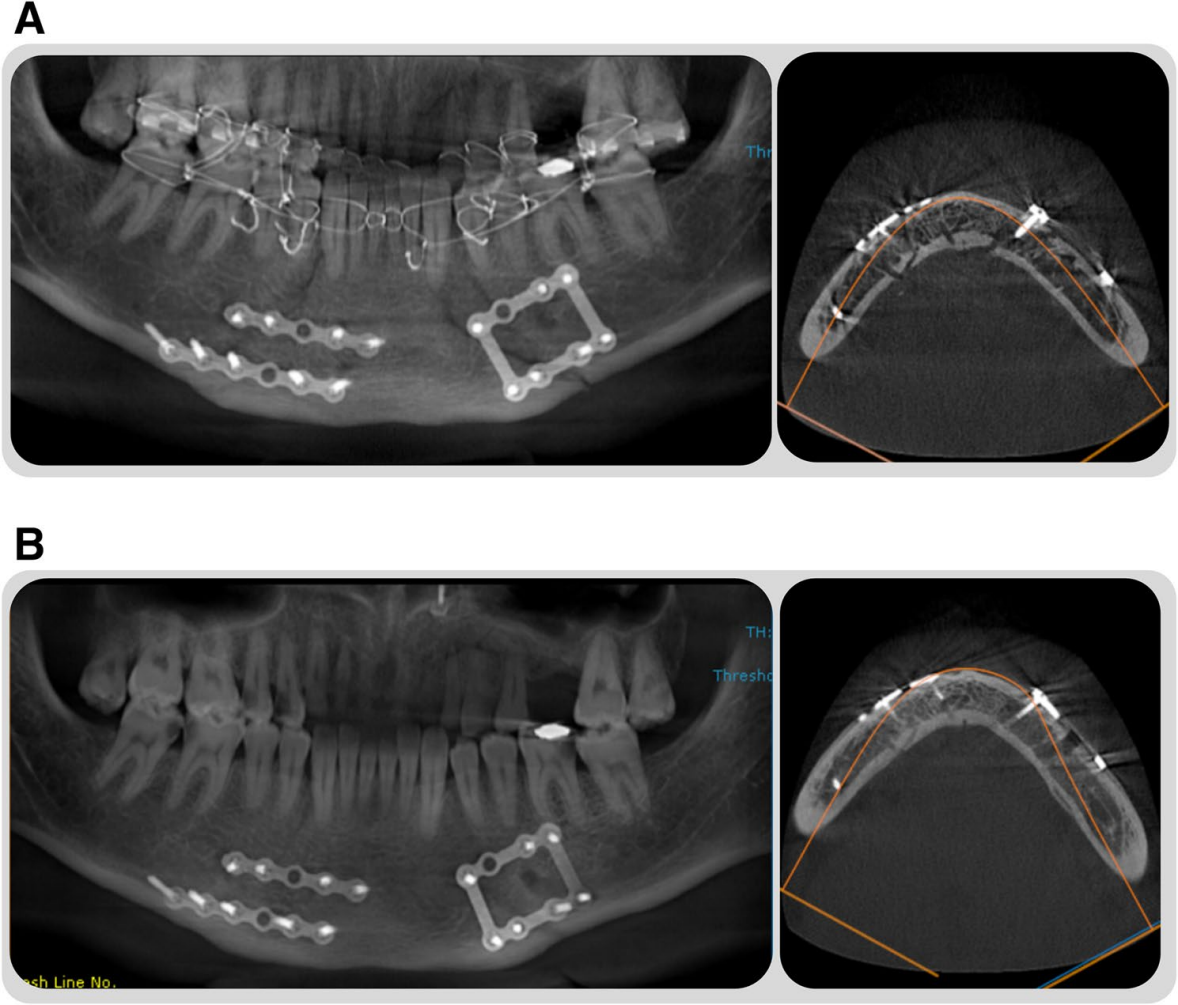
Postoperative radiographic assessment. **A**, Immediate postoperative CBCT scan. **B**, 12-week postoperative CBCT scan

An immediate Cone-Beam Computed Tomography (CBCT) scan was performed to evaluate the adequacy of fracture reduction and fixation (Fig. 5A), followed by a second scan at the 12th postoperative week (Fig. 5B) (Ingenuity Core; Philips Medical Systems, Cleveland, OH). The CBCT machine was standardized and phantom calibrated before each scan. Assessment of the mean bone density at the fracture line was performed in the 12-week scan, compared to the initial postoperative scan. Bone mineral density was measured on CBCT using OnDemand software (OnDemand 3D APP-DBM, Cybermed, Seoul, South Korea). Six 3*3 Region of Interest (ROI) were taken along the fracture line, and the mean value was calculated for each scan [6, 12]. Bone density values were expressed in Hounsfield Units (HU) after conversion from grayscale values [13].

### Statistical analysis

Data were fed to the computer and analyzed using IBM-SPSS software package V.20.0. (Armonk, NY: IBM Corp). The Shapiro-Wilk test was used to verify the normality of the distribution. The Paired t-test was utilized for normally distributed quantitative variables, while the Friedman test was opted for non-normally distributed quantitative variables and for ordinal data, with Post Hoc Test (Dunn’s) for pairwise comparisons. The Cochran’s test was used for a binary response variable, and the McNemar Test was used for categorical variables. The significance of the obtained results was judged at the 5% level.

## Results

The study was conducted on 12 patients suffering from bilateral parasymphyseal mandibular fractures in the MF zone. The Demographic data of the study are presented in Table 1. Regarding the postoperative pain assessment, a statistically significant decrease in pain was reported (*P* < 0.001), with complete resolution by the 6th week (Supplementary Table 1).

**Table 1:** Demographic data and epidemiology of the study (*P* = 12)

*P* = 12	No. (%)
Gender	
Male	11 (91.7%)
Female	1 (8.3%)
Age (years)	
Min. – Max	18–45
Mean ± SD	28.25 ± 8.44
EOT	
RTA	9 (75%)
Claimed Falls	2 (16.7%)
IPV	1 (8.3%)
Associated fractures	
No	8 (66.6%)
Zygomaticomaxillary Complex	2 (16.7%)
Zygomaticomaxillary Complex + Frontal	2 (16.7%)

*EOT* Etiology of Trauma, *RTA* Road Traffic Accident, *IPV* Inter Personal Violence

Interfragmentary mobility assessment was conducted at each fracture line for all of the enrolled patients. None of the fracture lines in the 3D-IMP group reported interfragmentary mobility across the clinical evaluation period (*P =* 1.000). On the other hand, one (8.3%) fracture line in the 2MPs side exhibited slight intra-fragmentary mobility at the second follow-up session (*P* = 0.392) (Supplementary Table 2). Owing to the reported instability in the 2MPs side, this patient consequently reported deranged occlusion evaluation in the second follow-up period. All of the remaining cases showed satisfactory occlusal analysis outcomes. The affected patient was managed with an extended 1-week IMF period, after which no mobility was detected in the 2MPs side. In the 4-week follow-up session, the IMF was released with a satisfactory occlusion outcome. Changes in the occlusal statues across the clinical evaluation period were statistically insignificant (*P* > 0.062) (Supplementary Table 3).

Regarding wound healing assessment, both groups exhibited consistent healing without significant variation over time, except for one patient, who suffered from wound dehiscence on both sides at the second follow-up session. Complete healing of the affected wound was achieved by the end of the 4th week. Intragroup wound healing variation across the clinical follow-up period was statistically insignificant (*P* = 0.392 for both groups) (Supplementary Table 4).

Regarding objective nerve testing, early intragroup differences were statistically insignificant (*P* = 0.096). In the second examination session, improvement of mental nerve sensation was reported in both groups, where 9 (75%) and 2 (16.7%) sides showed level B sensation in 3D-IMP and 2MPs sides, respectively. Despite that intragroup difference showed a statistically significant difference in the first postoperative week (*P* = 0.038^*^). By the end of the clinical examination period (6th week), a normal mental nerve response was reported in all the fractures in the 3D-IMP side (level A), while one side (8.3%) in the 2MPs-group was only responsive to noxious stimuli through thermal discrimination (TD) (level C) (4.17% of all the fractured lines in the study) (Table 2). Improvement in mental nerve sensation across the clinical follow-up period was statistically significant on both sides (*P* < 0.001^*^). No patients in the 3D-IMP group complained of parathesis at the end of the follow-up period, while one patient experienced numbness in the fractured side treated by conventional miniplates.

**Table 2:** Objective nerve testing analysis for the 3D-interlocking and conventional miniplates

**Objective Nerve testing **	**24hr**	**1** ^**st**^ ** week**	**4** ^**th**^ ** week**	**6** ^**th**^ ** week**	**Fr ** **(** **P** **)**
3D-IMP (S=12)					
An (0)	0 (0.0%)	0 (0.0%)	0 (0.0%)	0 (0.0%)	26.930^*^ <0.001^*^
C (1)	4 (33.3%)	1 (8.3%)	0 (0.0%)	0 (0.0%)	
B (2)	7 (58.3%)	9 (75.0%)	3 (25.0%)	0 (0.0%)	
A (3)	1 (8.3%)	2 (16.7%)	9 (75.0%)	12 (100.0%)	
**2-MPs (S=12)**					
An (0)	1 (8.3%)	1 (8.3%)	1 (8.3%)	0 (0.0%)	30.980^*^ <0.001^*^
C (1)	6 (50.0%)	4 (33.3%)	0 (0.0%)	1 (8.3%)	
B (2)	5 (41.7%)	7 (58.3%)	7 (58.3%)	0 (0.0%)	
A (3)	0 (0.0%)	0 (0.0%)	4 (33.3%)	11 (91.7%)	
**Z ** **(** **P** **)**	1.667 (0.096)	2.070^*^ (0.038^*^)	1.897 (0.058)	1.000 (0.317)	

*An* Anesthetic,*C* noxious stimuli and thermal discremination,*B* Proprioception,*A* normal nerve function, *3D-IMP* Side managed with 3D-Interlocking miniplates, *2-MPs* Side managed with conventional miniplates, *Fr* Friedman test,*Z* Wilcoxon signed rankstest, *S* Side, *SD* Standard deviation. *Statistically significant difference at *P*-value <0.05

Radiographic assessment of the adequacy of reduction and bone healing analysis via bone density measurement was conducted using immediate and 3-month postoperative CBCT scans. Postoperative scans showed successful reduction in all patients, with well-aligned mandibular bone structures. The reported mean bone density showed a statistically significant increase in the 3-month scan (*P* < 0.001^*^). While the Inter-group variation was insignificant in the immediate postoperative mean bone density (*P* = 0.195), the 3D-IMP group demonstrated significantly higher bone density at the 3-month scan (*P* < 0.001^*^) (Table 3).

**Table 3:** Bone density (Hu) analysis for the 3D-interlocking and conventional miniplates

Bone Density (Hu) (*P* = 12)			3D-IMP (S = 12)		2-MPs (S = 12)	t (*p*)	
Baseline Immediate Postoperative Scan	Mean ± SD.		678.6 ± 105.8		647.5 ± 97.52	1.380 (0.195)	
	Median.		694.9		653.4		
	Min – Max.		523.2–857.2		458.4–791.9		
3-month’ Postoperative Scan	Mean ± SD.		1107.4 ± 142.2		960.9 ± 97.58	4.266^*^ (0.001^*^)	
	Median.		1089.4		954.5		
	Min – Max.		855.0–1311.9		810.5–1102.0		
*t*			20.341*		27.200*		
*(p)*			(< 0.001^*^)		**(< 0.001** ^*^)		

*Hu* Hounsfield Units, *3D-IMP* Side managed with 3D-Interlocking miniplates, *2-MPs* Side managed with conventional miniplates, *t* Paired-t test for dependent varaibles, *S* Side, *SD* Standard deviation. *Statistically significant difference at *P*-value < 0.05

## Discussion

Fractures occurring within the mandibular parasymphyseal zone are conventionally managed using a dual miniplate fixation, which has generally demonstrated satisfactory stability of the fracture segments [1, 2]. Although 3D-plates have been shown to offer superior biomechanical performance, their application in oblique or body fractures involving the mental foramen remains contraindicated due to the potential risk of injury to the mental neurovascular bundle [14]. Hence, this split-mouth study was conducted to evaluate and compare the clinical and radiographic performance of 3D-Interlocking miniplates in the management of mandibular fractures near the MF.

Our study showed a predominance of male patients in their third decade of life, with an 11:1 male-to-female ratio and a mean age of 28.25 ± 8.44 years. This age group could be correlated to the predominance of road traffic accidents as the main trauma etiological factor in this study. A similar outcome was reported in the literature, especially in developing countries [14, 15]. The lack of obedience to traffic regulations and safety protocols, along with reckless driving, could contribute to the elevated rate of traumatic incidents, especially among the male adolescent demographic.

After the operation, none of the fractures in the 3D-IMP side had interfragmentary mobility, while 5.6% of fractures in the 2MPs side suffered interfragmentary mobility up to the 4th postoperative week. This was consistent with the findings of Xue et al., who demonstrated that 3D-plates have demonstrated superior initial interfragmentary stability when compared with the conventional miniplates in the Champy dictated configuration [16]. The strut-like design of 3D-plates offers enhanced biomechanical performance relative to earlier plating systems used in the management of mandibular fractures. A biomechanical research by Alkan et al. demonstrates that the box-shaped configuration of 3D-plates creates a broader platform that, unlike the linear screw placement on either side of the fracture line, increases resistance to torsional forces and thereby contributes to improved interfragmentary stability [17]. According to Vineeth et al., initial mobility was observed in 40% of cases treated with conventional miniplates compared to only 10% in those managed with 3D-plates following mandibular fracture fixation. In this study, all fracture lines demonstrated bimanual interfragmentary stability by the end of the first postoperative month. These findings suggest that, in contrast to linear plate systems, 3D-plates offer superior early primary stability [18].

In terms of the postoperative occlusion, one patient had a deranged occlusion in one side of the mandible owing to the fracture segments instability of the 2MPs side, resulting in a deranged intercuspation of teeth. On the other hand, normal intercuspation and satisfactory occlusion were attained in all of the 3D-IMP sides. This aligns with previous studies, which have reported occlusal disturbances in 0% to 20% of cases treated with 3D-plates [19]. The improved postoperative occlusion is largely attributed to the enhanced segmental stability provided by the structural design of 3D-plates. Supporting this, Barde et al. observed more favorable occlusal outcomes in patients managed with 3D plates compared to those treated with conventional miniplates [20].

The interfragmentary mobility and occlusal outcomes could be correlated and interpreted to the bone density analysis results in this study. A significant increase in bone density was observed in both groups over the 3 months (*P* < 0.001), with no notable difference between groups in the immediate postoperative phase (*P* = 0.195). However, in the 3-month scan, the 3D-IMP side exhibited significantly greater bone density compared to the 2MPs side (*P* < 0.001).

The implementation of the 3D-plate design in the MF zone allowed enhanced resistance to torsional forces and mechanical stability across the fracture line. This directly attributes to the lack of micromotion across the fracture line during the early healing period, leading to more effective bone regeneration across the fracture site. Sarepally et al. compared the clinical and radiographic performance of 3D-plates and 2.0 miniplates. Their outcome falls in line with that reported in this study, where an enhanced early bone healing could be associated with 3D miniplates fixation [20].

The radiographic analysis in this study was attained using a preoperative CT-scan, and two postoperative CBCT-scans within a 12-week timespan. The adoption of CBCT in the postoperative period for the combined purpose of achieving a reliable bone density analysis while at the same time adhering to the radiographic doctrines of unjustified extra radiation usage [13]. The utilization of CBCT for bone density assessment has proven to be a reliable method, yet several steps are required in order to achieve reproducible results. Standardization of the CBCT acquisition machine, with a similar field of view, phantom calibration, and software configuration, is of paramount importance. In this report, all of the above-mentioned practices were conducted in order to achieve a reliable outcome.

The conceived design allowed the utilization of the added benefits of the 3D-plates while preserving the integrity of the mental nerve. To accommodate anatomical variability, a range of 3D-IMP with different U-shaped vertical bar lengths (6–9 mm) was pre-fabricated and available before surgery. The appropriate plate is selected based on a thorough analysis of preoperative CT imaging.

Sensory nerve function was evaluated in this study using two methods: a subjective test, which depends on the patient’s sensation of paresthesia. Objective neurosensory assessment was conducted following a modified version of the Zuniga and Essick protocol [8]. Both groups showed statistically significant improvement in the 6th week postoperatively (*p* < 0.001), giving a positive response to level A testing, with the exception of one patient who was unresponsive in the fracture side treated by 2MPs until the 4th week before ultimately reacting to thermal testing (level C) at the end of the follow-up period. This might have been caused by severe pre-operative mobility of the fractured segments in this affected side, leading to a detrimental effect on neurosensory recovery. Neurosensory recovery has a plethora of influencing factors. Mundepi et al. demonstrate that severe displacement at the fracture line of more than 5 mm could lead to a delayed functional neurosensory recovery [21]. The Zuniga and Essick protocol was used to evaluate neurosensory performance instead of the commonly used quantitative sensory testing. The electro-physiological testing method utilizes the healthy side as a baseline for the affected side. This was unapplicable in our study due to the involvement of both sides of the mandible. The neurosensory outcomes in this study reported full regain in the normal nerve conduction, bar for one side that showed a response to thermal testing (level C). Although promising, the outcome may point to the need for a longer follow-up period for the assessment of the neurosensory recovery.

Both groups showed comparable subjective and objective nerve testing results. A statistically significant early improvement is reported in the 3D-IMP side at the first postoperative week (*P* = 0.038^*^). Kulkarni et al. report that the majority of nerve recovery occurs within the initial 4 months after fracture management [8]. The improved outcome in this study could be attributed to the utilized nerve assessment methodology. The Zuniga and Essick protocol is a qualitative descriptive objective tool that allows grading of the degree of patient bothersome after daily normal function or behavior. The split-mouth nature of this study prevented the utilization of electric nerve testing methodology owing to the lack of a control contralateral side.

Zuniga reports that therapeutic intervention is usually sufficient to manage neuropathic pain following mandibular fractures [22]. Nerve skeletonization was attained for proper fracture line plating in both groups; however, in this study, the 3D-IMP required less nerve dissection. This is related to our experience and improved learning curve from the application of 3D and interlocking miniplates [6]. Cillo et al. report the complete functional neurosensory recovery of all their subjects where mental nerve skeletonization was performed for intraoral internal fixation [23].

In terms of wound healing assessment, both groups demonstrated consistent and uneventful healing throughout the follow-up period, with no significant intragroup differences. Liu et al. reported that the incidence of postoperative complications such as wound dehiscence, malocclusion, and hardware failure was significantly lower with three-dimensional plates compared to conventional miniplates [24]. In our study, no cases of screw loosening or plate fracture were observed in either group. For the sake of standardization, both sides were managed with mandibular 2.0-miniplates with a similar 1.5 profile. The overlapping feature of the interlocking plate allowed a uniform thickness across the plate. This prohibited any unnecessary added thickness, especially in the upper border of the plate near the incision line. This could explain the favorable clinical wound healing outcomes reported in this study.

The split-mouth nature of this study allowed for the curbing of the confounding factors that could affect the results of the utilized fixation techniques. The known carry-over effect of these self-controlled studies was evident in the occlusion analysis in this study. Furthermore, the lack of a washout period in this split-mouth design could add inaccuracies in the pain analysis. The relatively short follow-up period may be insufficient to fully capture long-term neurosensory recovery. Blinding of the operating surgeon was not feasible due to the nature of the intervention; however, selection bias was evaded by the means of rigorous allocation concealment practice. Patient blinding and the split-mouth design helped minimize subjective and inter-individual confounding factors. In addition, the small cohort size of the study might affect the general distribution of the outcomes. Larger randomized trials with longer follow-up duration are required to confirm the present findings.

The ability to fit the interlocking plate to the nature, height, and curvature of each patient may also be pointed out as one of the limitations in the surgical technique. Additionally, careful consideration must be given to the length and orientation of dental roots when determining both the optimal plate length and the angulation of screw placement. However, this is a recurring complication in any direct internal fixation modality.

## Conclusion

The excellent clinical and radiographic results observed in this study suggest that the use of the 3D-Interlocking mini plates in mandibular fractures around the mental foramen is a practical alternative to the conventional mini plates, offering enhanced torsional force resistance and promoting better bone healing while maintaining the integrity of the neurovascular bundle.

## Supplementary Information

Supplementary Material 1.

## Data Availability

All data generated or analysed during this study are included in this published article.

## Abbreviations

MF: : Mental foramen
3D-IMP: Three-dimensional
CT: Computed tomography
b.i.d: Twice in the day
SLT: Static light touch
TPD: Two-point discrimination
HU: Hounsfield units
SNOSE: Sequentially numbered, opaque, sealed envelopes
3D: Three-dimensional
2MPs: Conventional double miniplates
IMF: Intermaxillary fixation
VAS: Visual analogue scale
BDS: Brush directional stroke
TD: Thermal discrimination
CBCT: Cone-beam computed tomography
