# Supplementary material for: Three-dimensional Interlocking versus conventional miniplates for mandibular fractures fixation near the mental foramen: a split-mouth randomized clinical trial
Source: BMC Oral Health. 2026 Mar 29;26:709. doi: 10.1186/s12903-026-07799-5 (PMC13101217; doi:10.1186/s12903-026-07799-5)
Supplement: Supplementary file 1 — Supplementary Material 1. [file 12903_2026_7799_MOESM1_ESM.docx]

| **Supplementary Table 1: Pain analysis for the enrolled patients** | | | | | | |
| --- | --- | --- | --- | --- | --- | --- |
| ***VAS*** | **24hr** | **1^st^ week** | **4^th^ week** | **6^th^ week** | **Fr**  **(*P*)** | |
|  | | | | | | |
| **(P=12)** |  |  |  |  |  | |
| Min. – Max. | 2.0 – 9.0 | 1.0 – 7.0 | 0.0 – 4.0 | 0.0 – 0.0 | **35.053^*^**  **(<0.001^*)^** | |
| Mean ± SD. | 5.25 ± 2.05 | 3.17 ± 1.95 | 1.17 ± 1.47 | 0.0 ± 0.0 |  |  |
| Median (IQR) | 5.0 (4.0 – 6.50) | 2.50 (2.0 – 4.50) | 0.50 (0.0 – 2.50) | 0.0 (0.0 – 0.0) |  |  |
|  | | | | | | |
| **(*P^1^*)** |  | 0.058 | **<0.001*** | **<0.001*** |  |  |
| **VAS:** Visual Analogue Score**; Fr**: Friedman test; **P**: Patient; **P^1^ :** p value for comparing between **24hr** and each other periods  **SD**: Standard deviation;*****Statistically significant difference at *P*-value <0.05. | | | | | | |

| **Supplementary Table 2: Analysis of interfragmentary mobility for the 3D-interlocking and conventional miniplates** | | | | | |
| --- | --- | --- | --- | --- | --- |
| ***Interfragmentary Mobility*** | **24hr** | **1^st^ week** | **4^th^ week** | **6^th^ week** | **Fr**  **(*P*)** |
|  | | | | | |
| **3D-IMP (S=12)** |  |  |  |  |  |
| No mobility | 12 (100.0%) | 12 (100.0%) | 12 (100.0%) | 12 (100.0%) | (1.000) |
| Slight mobility | 0 (0.0%) | 0 (0.0%) | 0 (0.0%) | 0 (0.0%) |  |
| Moderate mobility | 0 (0.0%) | 0 (0.0%) | 0 (0.0%) | 0 (0.0%) |  |
| Severe mobility | 0 (0.0%) | 0 (0.0%) | 0 (0.0%) | 0 (0.0%) |  |
|  | | | | | |
|  | | | | | |
| **2-MPs (S=12)** |  |  |  |  |  |
| No mobility | 11 (91.7%) | 11 (91.7%) | 12 (100.0%) | 12 (100.0%) | (0.392) |
| Slight mobility | 1 (8.3%) | 1 (8.3%) | 0 (0.0%) | 0 (0.0%) |  |
| Moderate mobility | 0 (0.0%) | 0 (0.0%) | 0 (0.0%) | 0 (0.0%) |  |
| Severe mobility | 0 (0.0%) | 0 (0.0%) | 0 (0.0%) | 0 (0.0%) |  |
|  | | | | | |
| **3D-IMP**: Side managed with 3D-Interlocking miniplates; **2-MPs**: Side managed with conventional miniplates; **Fr**: Fridman test; **Z:** Wilcoxon signed ranks test ; **S**: Side; **SD**: Standard deviation;*****Statistically significant difference at *P*-value <0.05. | | | | | |

| **Supplementary Table 3: Occlusion analysis for the enrolled patients** | | | | | |
| --- | --- | --- | --- | --- | --- |
| ***Occlusion*** | **24hr** | **1^st^ week** | **4^th^ week** | **6^th^ week** | **Fr**  **(*P*)** |
|  | | | | | |
| **(P=12)** |  |  |  |  |  |
| Stable | 11 (91.7%) | 11 (91.7%) | 12 (100.0%) | 12 (100.0%) | (0.062) |
| Mild unstable | 1 (8.3%) | 1 (8.3%) | 0 (0.0%) | 0 (0.0%) |  |
| Moderately unstable | 0 (0.0%) | 0 (0.0%) | 0 (0.0%) | 0 (0.0%) |  |
| Severely unstable | 0 (0.0%) | 0 (0.0%) | 0 (0.0%) | 0 (0.0%) |  |
|  | | | | | |
| **Fr**: Friedman test; **P**: Patient; **SD**: Standard deviation;*****Statistically significant difference at *P*-value <0.05. | | | | | |

| **Supplementary Table 4: Incidence of wound dehiscence occurrence for the 3D-interlocking and conventional miniplates** | | | | | |
| --- | --- | --- | --- | --- | --- |
| ***Wound Dehiscence*** | **24hr** | **1^st^ week** | **4^th^ week** | **6^th^ week** | **Q**  **(*P*)** |
|  | | | | | |
| **3D-IMP (S=12)** |  |  |  |  |  |
| No . | 11 (91.7%) | 11 (91.7%) | 12 (100.0%) | 12 (100.0%) | 3.00  )0.392( |
| Yes. | 1 (8.3%) | 1 (8.3%) | 0 (0.0%) | 0 (0.0%) |  |
|  | | | | | |
|  | | | | | |
| **2-MPs (S=12)** |  |  |  |  |  |
| No . | 11 (91.7%) | 11 (91.7%) | 12 (100%) | 12 (100.0%) | 3.00  )0.392( |
| Yes. | 1 (8.3%) | 1(8.3%) | 0 (0.0%) | 0 (0.0%) |  |
|  | | | | | |
| **3D-IMP**: Side managed with 3D-Interlocking miniplates; **2-MPs**: Side managed with conventional miniplates; **Q**: Cochran's test; **S**: Side; **SD**: Standard deviation;*****Statistically significant difference at *P*-value <0.05. | | | | | |
